# Tetracycline accelerates the temporally-regulated invasion response in specific isolates of multidrug-resistant *Salmonella enterica* serovar Typhimurium

**DOI:** 10.1186/1471-2180-13-202

**Published:** 2013-09-11

**Authors:** Brian W Brunelle, Shawn MD Bearson, Bradley L Bearson

**Affiliations:** 1 Food Safety and Enteric Pathogens Research Unit, National Animal Disease Center, ARS, USDA, Ames, IA 50010, USA; 2 Agroecosystems Management Research Unit, National Laboratory for Agriculture and the Environment, ARS, USDA, Ames, IA 50010, USA

**Keywords:** Antibiotics, Drug-resistant, Invasion, Salmonella, Tetracycline, Typhimurium, DT104, DT193

## Abstract

**Background:**

Multidrug-resistant (MDR) *Salmonella* isolates are associated with increased morbidity compared to antibiotic-sensitive strains and are an important health and safety concern in both humans and animals. *Salmonella enterica* serovar Typhimurium is a prevalent cause of foodborne disease, and a considerable number of *S*. Typhimurium isolates from humans and livestock are resistant to three or more antibiotics. The majority of these MDR *S*. Typhimurium isolates are resistant to tetracycline, a commonly used and clinically and agriculturally relevant antibiotic. Because exposure of drug-resistant bacteria to antibiotics can affect cellular processes associated with virulence, such as invasion, we investigated the effect tetracycline had on the invasiveness of tetracycline-resistant MDR *S.* Typhimurium isolates.

**Results:**

The isolates selected and tested were from two common definitive phage types of *S.* Typhimurium, DT104 and DT193, and were resistant to tetracycline and at least three other antibiotics. Although *Salmonella* invasiveness is temporally regulated and normally occurs during late-log growth phase, tetracycline exposure induced the full invasive phenotype in a cell culture assay during early-log growth in several DT193 isolates. No changes in invasiveness due to tetracycline exposure occurred in the DT104 isolates during early-log growth or in any of the isolates during late-log growth. Real-time PCR was used to test expression of the virulence genes *hilA*, *prgH*, and *invF*, and these genes were significantly up-regulated during early-log growth in most isolates due to tetracycline exposure; however, increased virulence gene expression did not always correspond with increased invasion, and therefore was not an accurate indicator of elevated invasiveness. This is the first report to assess DT193 isolates, as well as the early-log growth phase, in response to tetracycline exposure, and it was the combination of both parameters that was necessary to observe the induced invasion phenotype.

**Conclusions:**

In this report, we demonstrate that the invasiveness of MDR *S.* Typhimurium can be modulated in the presence of tetracycline, and this effect is dependent on growth phase, antibiotic concentration, and strain background. Identifying the conditions necessary to establish an invasive phenotype is important to elucidate the underlying factors associated with increased virulence of MDR *Salmonella*.

## Background

*Salmonella* is the most common cause of bacterial food-borne illness in the U.S. and is estimated to annually cause over 1 million cases, 19,000 hospitalizations, 350 deaths, and $2.6 billion in social costs [1,2]. *Salmonella enterica* serovar Typhimurium is one the most prevalent salmonellae in humans and livestock, and many of these cases are found to be resistant to multiple antibiotics. According to the National Antimicrobial Resistance Monitoring System (NARMS), 27-83% of *S.* Typhimurium isolates from humans, chicken, cattle, and swine were found to be resistant to three or more classes of antibiotics [3]. A recent *Salmonella* Typhimurium isolate linked to an outbreak associated with ground beef was resistant to eight antibiotics: amoxicillin/clavulanic acid, ampicillin, ceftriaxone, cefoxitin, kanamycin, streptomycin, sulfisoxazole, and tetracycline [4].

Multidrug-resistant (MDR) *Salmonella* is associated with increased morbidity in humans and increased mortality in cattle relative to sensitive strains [5,6]. There are several non-exclusive rationales for these clinical observations [7,8]. One explanation is treatment failure, where the administered antibiotic is ineffective due to bacterial resistance, and therefore the infection persists and the illness progresses. Another explanation is that the normal gut flora is disrupted by an antibiotic regimen, thereby increasing the risk of an opportunistic infection by drug-resistant bacteria. Finally, there is the possibility that antibiotics can directly enhance bacterial virulence; this concept is supported by several publications reporting that certain antibiotics can alter virulence factors in some bacteria *in vitro*[9-12], including tetracycline in *S.* Typhimurium definitive phage type DT104 [13]. However, the report by Weir et al. tested a single DT104 isolate at a single tetracycline concentration during late-log growth and identified a significant change in virulence gene expression, while an earlier report by Carlson et al. evaluated over 400 DT104 isolates exposed to tetracycline that were grown to stationary phase and did not observe any isolates with a significantly increased ability to invade cells in culture [14]. Resistance to tetracycline is prominent among *S.* Typhimurium isolates in humans (34%), chickens (39%), cattle (59%), and swine (88%) according to a ten-year average from the National Antimicrobial Resistance Monitoring System [3,15]; thus, our objective was to explore the relationship between gene expression and cellular invasion in response to tetracycline. We examined the effects of sub-inhibitory tetracycline concentrations on isolates of phage type DT104 and DT193 during early-log and late-log growth to determine the conditions, if any, that affect MDR *Salmonella* Typhimurium invasiveness after tetracycline exposure. We ascertained that an induced-invasion phenotype was a dose-dependent response due to the combination of two novel study parameters, early-log growth and DT193 isolates. We also found that expression of virulence genes can be tetracycline-induced during either early-log or late-log growth in many isolates, but this did not always correlate with increased invasiveness.

## Results

### Selection of isolates

A total of forty *S.* Typhimurium DT104 and DT193 isolates from cattle were characterized for resistance to ampicillin, chloramphenicol, gentamicin, kanamycin, streptomycin, and tetracycline. Isolates resistant to tetracycline and at least three additional antibiotics, but sensitive to gentamicin (which is needed to kill extracellular bacteria in the invasion assays), were then screened for the presence of the *Salmonella* genomic island 1 (SGI-1) and tetracycline resistance genes known to occur in *Salmonella* (*tetA*, *B*, *C*, *D*, and *G*). The SGI-1 is a 43 kb stable chromosomal integron often found in DT104, and it encodes several antibiotic resistance genes as well as hypothetical genes that have a potential association with virulence [16-18]. The SGI-1 was identified in all DT104 isolates but in none of the DT193 isolates. All the DT104 isolates encoded a single tetracycline resistance gene, *tetG*, while the DT193 isolates encoded the following combinations: *tetA*; *tetA, B, C,* and *D*; or *tetB, C,* and *D*. Representatives of each *tet*-resistance gene combination were selected at random for further study (Table 1).

**Table 1:** **Characterization of antibiotic resistance profiles and tetracycline resistance genes in eight *S. *typhimurium isolates**

**Isolate**	**Phagetype**	**Resistance profile**						***tet *gene(s)**				
		**amp**	**chlor**	**gent**	**kan**	**strp**	**tet**	***tetA***	***tetB***	***tetC***	***tetD***	***tetG***
1434	DT193	**+**	**+**	-	**+**	**+**	**+**	**+**	-	-	-	-
5317	DT193	**+**	**+**	-	**+**	**+**	**+**	**+**	-	-	-	-
752	DT193	**+**	**+**	-	-	**+**	**+**	**+**	-	-	-	-
1306	DT193	**+**	**+**	-	**+**	**+**	**+**	**+**	**+**	**+**	**+**	-
4584	DT193	**+**	**+**	-	**+**	**+**	**+**	-	**+**	**+**	**+**	-
530	DT104	**+**	**+**	-	**-**	**+**	**+**	-	-	-	-	**+**
290	DT104	**+**	**+**	-	**+**	**+**	**+**	-	-	-	-	**+**
360	DT104	**+**	**+**	-	**-**	**+**	**+**	-	-	-	-	**+**

### Selection of antibiotic concentrations

**Figure 1:**
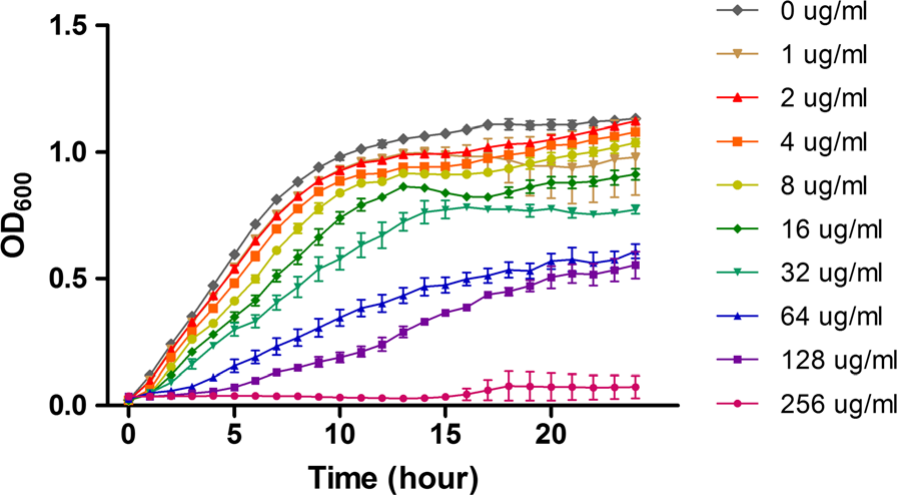
**Representative growth curve of multidrug-resistant *S*. Typhimurium exposed to various concentrations of tetracycline.** Serial two-fold dilutions of tetracycline (0–256 μg/ml) were added at OD_600_ = 0.15 to each of the eight isolates to determine the effect of tetracycline exposure on growth. The growth curve of isolate 1434 is shown.

Growth curves were determined for each of the eight isolates over a range of tetracycline concentrations (0–256 μg/ml). The growth curve for isolate 1434, which is representative of all the isolates, is shown in Figure 1. Tetracycline concentrations between 1–128 μg/ml did not prevent growth, and this range was considered sub-inhibitory for this study. No significant change in growth due to antibiotic addition was observed between 1–32 μg/ml of tetracycline. Subsequent invasion and gene expression analyses were performed using several concentrations of tetracycline within this range (0, 1, 4, and 16 μg/ml) in order to assess if an effect on invasion was concentration dependent.

### Tetracycline induces invasion in a subset of isolates during early-log phase

**Figure 2:**
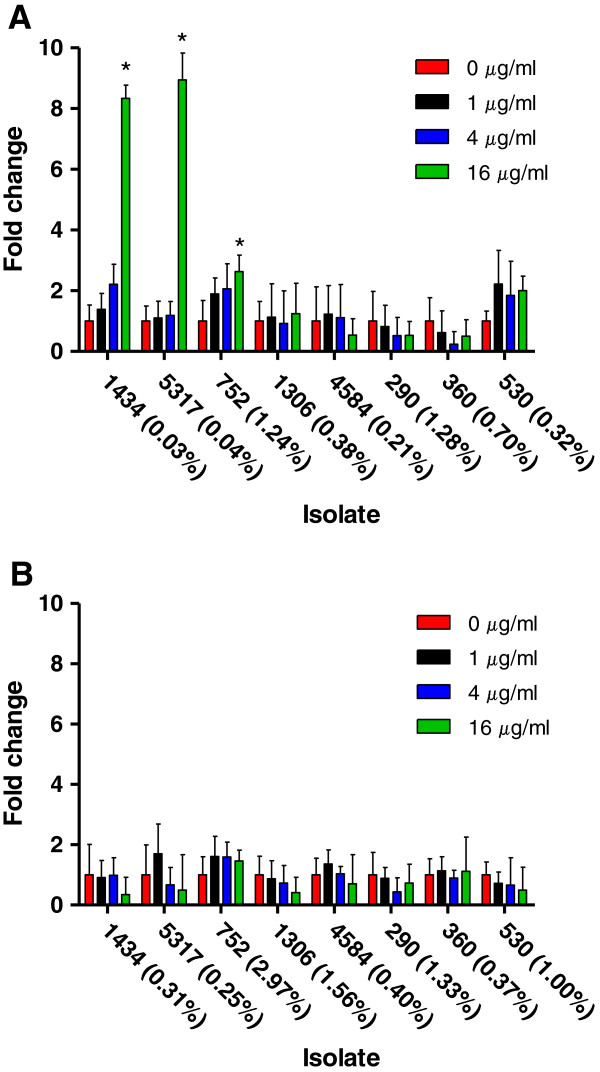
**Changes in *S. *Typhimurium invasiveness at early- and late-log growth after tetracycline exposure.** Invasion assays were performed on *S.* Typhimurium isolates grown to either early- or late-log phase and exposed to four different tetracycline concentrations (0, 1, 4, and 16 μg/ml) for 30 minutes. Changes in invasion were normalized to the control dose (0 μg/ml) for each isolate at **(A)** early-log and **(B)** late-log growth phase. The “*” indicates a significant change based on the pre-normalized data. The numbers in parentheses indicate percent invasion at the control dose (0 μg/ml) for each isolate.

Regulation of the invasion process is initiated during early-log phase of growth [19], and *Salmonella* becomes fully invasive during the late-log phase [20]. Cellular invasion assays were performed using isolates grown to early-log phase (OD_600_ = 0.15) and exposed to 0, 1, 4, and 16 μg/ml of tetracycline for 30 minutes to address the effect tetracycline had on *Salmonella* invasiveness (Figure 2A; Additional file 1). Three DT193 isolates (1434, 5317, and 752) had a significant increase in invasion during early-log growth in the presence of 16 μg/ml tetracycline, and all three of these isolates have in common the presence of a single tetracycline resistance gene, *tetA* (Table 1). Tetracycline exposure did not enhance the invasion phenotype of the other DT193 isolates or the three DT104 isolates.

To determine if tetracycline exposure enhances *Salmonella* invasiveness during late-log phase, isolates were grown to OD_600_ = 0.60 and exposed to 0, 1, 4, and 16 μg/ml of tetracycline for 30 minutes. Tetracycline did not increase the invasiveness of *Salmonella* during late-log growth in any of the isolates (Figure 2B; Additional file 1). However, the level of invasion induced by 16 μg/ml tetracycline during early-log phase in the three DT193 isolates was similar to the invasion levels of their respective controls (0 μg/ml) during late-log phase. These results demonstrate that when *Salmonella* is at its highest level of normal invasion (late-log), exposure to sub-inhibitory levels of tetracycline does not result in hyperinvasiveness; instead, tetracycline exposure triggers the invasive phenotype in specific isolates during a phase of growth that *Salmonella* is not otherwise fully invasive (early-log).

### Gene expression changes due to tetracycline exposure

**Figure 3:**
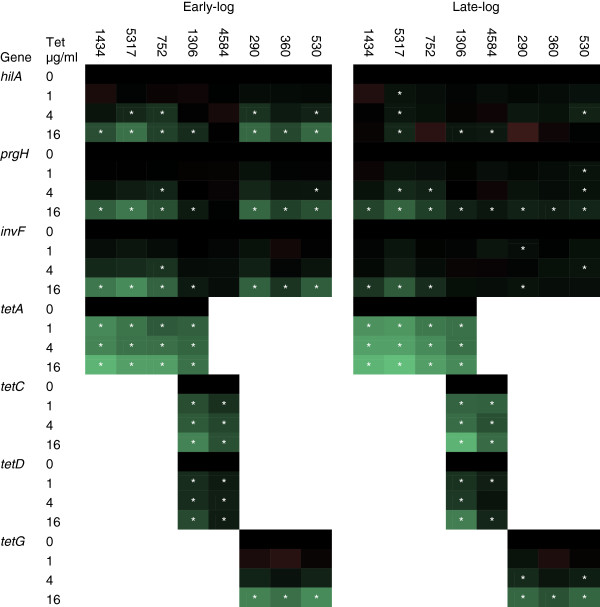
**Gene expression changes in *S. *Typhimurium at early- and late-log growth after tetracycline exposure.** Real-time gene expression assays were performed on *S.* Typhimurium isolates grown to either early- or late-log phase and exposed to four different tetracycline concentrations (0, 1, 4, and 16 μg/ml) for 30 minutes. Virulence genes (*hilA*, *prgH*, and *invF*) and tetracycline resistance genes (*tetA*, *B*, *C*, *D*, and *G*) were profiled. Compared to the control for each gene (0 μg/ml), black indicates no gene expression change, green indicates an increase in gene expression, and red indicates a decrease in gene expression; the brighter the green or red, the greater the change. The white “*” denotes a significant change in expression compared to the control.

The relative transcript levels of three genes associated with invasion regulation (*hilA*, *prgH*, and *invF*), as well as the tetracycline resistance genes in each isolate (*tetA*, *B*, *C*, *D*, and/or *G*), were determined by real-time PCR. The *hilA* gene is essential for invasion as HilA activity regulates downstream invasion factors, which includes the *prgH* and *invF* genes [21,22]. Together, these genes provide a direct and indirect measure of both the *hilA* transcript and HilA protein, respectively. During early-log phase, all three invasion genes were significantly up-regulated in seven of the eight isolates at 16 μg/ml compared to the 0 μg/ml control, while four isolates had one or more of the invasion genes significantly up-regulated at 4 μg/ml; no invasion gene expression changes occurred in any isolate at 1 μg/ml (Figure 3; Additional file 1). The *tetA*, *tetC*, and *tetD* transcripts were significantly up-regulated during early-log in their respective isolates at all tetracycline concentrations (1, 4, and 16 μg/ml), while *tetG* was only significantly up-regulated at 16 μg/ml. No transcript was detected for *tetB* in the two isolates that encoded this gene. The *tetA, C,* and *D* genes were up-regulated at a concentration as low as 1 μg/ml tetracycline, whereas increased invasion gene expression occurred starting at 4 μg/ml, indicating changes in virulence factor gene expression due to tetracycline is dose-dependent. It should be noted that while 1 μg/ml is low for tetracycline resistant strains of *Salmonella*, it is inhibitory for sensitive strains.

During late-log phase, a significant increase in *hilA*, *prgH*, and/or *invF* expression was observed in response to tetracycline exposure in several isolates (Figure 3; Additional file 1). The effect of tetracycline on the *tet* genes was similar to the early-log data whereby *tetA, C,* and *D* were up-regulated starting at 1 μg/ml, though none of the *tetG* genes were up-regulated at this dose. Again, an increase in virulence gene expression was dependent on tetracycline concentration but did not coincide with increased invasiveness.

## Discussion

Multidrug-resistant *Salmonella* Typhimurium is a prevalent food safety and public health concern. Due to the fact that tetracycline resistance is frequently found in *S.* Typhimurium isolates from humans and livestock [3,15], our goal was to test and characterize the conditions necessary to generate an invasive phenotype in MDR *Salmonella* following tetracycline exposure. Two common MDR *S*. Typhimurium phage types are DT104 and DT193, and these are typically resistant to three or more antibiotics, are found in humans and livestock, and have been associated with foodborne outbreaks [23-27]. DT104 and DT193 share a similar antibiotic resistance profile, but the genetics underlying their resistance phenotype differ. For instance, the majority of resistance genes in DT104 isolates reside in the *Salmonella* genomic island 1 on the chromosome, whereas the resistance genes of DT193 are typically encoded on plasmids. Also, DT104 isolates generally have only the *tetG* gene to confer resistance to tetracycline, but isolates of DT193 can have a combination of *tetA-D* genes and usually lack *tetG*[28,29]. Since tetracycline is used therapeutically in humans and animals, and because most MDR *S*. Typhimurium isolates are resistant to tetracycline, our goal was to determine the effect and extent tetracycline exposure had on the invasiveness of *Salmonella* isolates from DT104 and DT193. We examined both cell culture invasion and virulence gene expression *in vitro* in response to tetracycline under a combination of three conditions: growth phase, tetracycline resistance genotype, and antibiotic concentration.

Cellular invasion is a temporally-regulated process in *Salmonella* that involves the activation of a sequence of genes, most importantly, *hilA*[21]. The *hilA* gene is the bottleneck in the process and its deletion prevents invasion from occurring, whereas its over-expression usually results in increased invasion [22]. The invasive response is initiated during early-log growth, and *Salmonella* is considered maximally invasive during the late-log growth phase [20]. We found that during early-log growth, tetracycline significantly increased cellular invasion in three isolates, while it significantly up-regulated the gene expression of virulence factors *hilA*, *prgH*, and *invF* in seven isolates. None of the isolates in the study had an increase in cellular invasion during late-log growth in response to tetracycline, but expression of virulence factors was up-regulated in several isolates. The increased invasiveness of the isolates during early-log was commensurate with the temporally-regulated invasive phenotype observed in each respective 0 μg/ml control isolate during late-log. Therefore, tetracycline exposure induced a shift in the invasion response to an earlier time in the growth cycle (early-log), yet tetracycline did not enhance invasion efficacy when invasion was already at its maximum potential in late-log growth. In addition, an increase in virulence gene expression did not always correlate with a reciprocal increase in invasion. The data demonstrates that the induction of invasion by tetracycline is a growth phase dependent response.

Several tetracycline concentrations were evaluated to determine if invasion induction was dependent on dose, or if the presence of tetracycline at any level would be effective. Three concentrations of tetracycline that did not inhibit growth of any of the isolates were chosen to study (1, 4, 16 μg/ml). The tetracycline-induced invasion response in the three isolates was only observed at 16 μg/ml. The induction of invasion by tetracycline is a dose dependent response.

DT104 and DT193 isolates that encode tetracycline resistance genes commonly found in *S*. Typhimurium (*tetA, B, C, D,* and *G*) were evaluated. The DT104 isolates all had SGI-1 and *tetG*, but no other tetracycline resistance genes were present. None of the DT193 isolates contained SGI-1. Of the five DT193 isolates, three had only a *tetA* gene, one had *tetA, B, C,* and *D*, and one had *tetB, C,* and *D*. Only the three DT193 isolates encoding a single tetracycline resistance gene, *tetA*, were more invasive during early-log growth at 16 μg/ml of tetracycline; the isolate that encoded *tetA-D* did not have this tetracycline-induced phenotype. All isolates, except the isolate encoding *tetB-D* (4584), had increased invasion gene expression following tetracycline exposure during early-log phase. Though a specific unknown mechanism that induces invasion in response to tetracycline may exist, it is not shared by all isolates and is independent of SGI-1. Induction of invasion due to tetracycline exposure is restricted to a subset of MDR *S*. Typhimurium isolates.

Previous work by Carlson et al. tested over 400 DT104 isolates that were exposed to tetracycline and grown to stationary phase, but no difference in invasion due to antibiotic treatment was observed [14]. Our data for the DT104 and DT193 isolates grown to late-log phase and then exposed to tetracycline are consistent with these results. Also, the increase in virulence gene expression during late-log growth after tetracycline exposure reported by Weir et al. [13] parallels our expression data. However, no previous study examined the effect of any antibiotic on DT193 or during early-log growth, and it was these two factors that were critical to observing the induction of the invasion phenotype due to tetracycline. The basis for the difference in response between DT193 and DT104 could be genetic content (e.g. the presence of additional virulence genes), the differential regulation of a particular response, or both.

Many studies have shown that antibiotics can directly or indirectly effect transcription and regulation of cellular processes [30-33]. In the current study, tetracycline up-regulated genes associated with virulence, but this was not always coincident with an increase in the invasive phenotype. The regulation of invasion is a complex network of interactions and responses, and it is possible that the tetracycline stimulus could affect targets downstream of *hilA*, *invF*, and *prgH*; such a response could up-regulate a repressor of invasion in the non-induced isolates. Genome sequencing of the isolates, plus transcriptomic analyses, will provide a more complete picture of what genes and processes are being affected by tetracycline exposure. Evaluation of other antibiotics would also discern if the response is specific to tetracycline, or if it is general to an antibiotic stress.

The response to tetracycline by some MDR *S*. Typhimurium isolates could provide a selective advantage to the bacteria by quickly and efficiently promoting entry into an intracellular niche within the host. Additionally, the use of efflux pumps to maintain viability in the presence of tetracycline is an active transport mechanism that requires energy to generate the proton gradient needed to drive the antiporter [34]; escaping such an environment would benefit the bacteria as fewer resources are required in the absence of the antibiotic. MDR *S*. Typhimurium already has a competitive advantage for survival due to its antimicrobial resistance phenotypes compared to sensitive strains, and our investigation identified an additional advantage of a tetracycline-inducible invasion phenotype that could influence colonization potential. Perhaps these factors are associated with the increased morbidity observed among MDR *Salmonella* patients.

## Conclusions

We have found that tetracycline can induce invasion in MDR *S.* Typhimurium, and that this response is dependent on antibiotic concentration, growth phase, and isolate. It does not appear that the induction of *Salmonella* invasiveness is a universal phenotypic response, even though the majority of isolates had an increase in virulence gene expression; a significant increase in *hilA* gene expression was not an accurate indicator of increased cellular invasion. Knowledge of the parameters necessary to establish this phenotype is important to further elucidate the underlying factors associated with increased virulence of MDR *Salmonella*.

## Methods

### Antibiotic-resistant profiles

Forty isolates of *Salmonella* Typhimurium phage types DT104 and DT193 originally collected from cattle were selected at random for antibiotic-resistance characterization from our NADC strain library. We defined drug-resistance by the presence of growth after culturing all isolates on separate LB plates overnight containing the following antibiotics and concentrations: ampicillin (100 μg/ml), chloramphenicol (30 μg/ml), gentamicin (100 μg/ml), kanamycin (50 μg/ml), streptomycin (100 μg/ml), or tetracycline (15 μg/ml). These cutoffs were adapted based on studies and prior experience with *Salmonella* grown in LB media [35-37], and all are near or above the CLSI breakpoint concentrations for ampicillin (32 μg/ml), chloramphenicol (32 μg/ml), gentamicin (16 μg/ml), kanamycin (64 μg/ml), streptomycin (64 μg/ml), and tetracycline (16 μg/ml).

### Characterization of *tet* resistance genes

Primers specific to *tetA*, *B*, *C*, *D*, and *G* genes were used to identify the tetracycline resistance gene(s) present in select isolates (Table 2); these are the tetracycline genes commonly observed in *Salmonella*[34]. Presence or absence of the *Salmonella* genomic island 1 (SGI-1) was detected with primers to the 5′ insertion site (*thdF*-S001), the internal S013 gene, and the most 3′ SGI-1 gene, S044 (Table 2). DNA was obtained by boiling a single colony from each isolate in 30 μl water. Each 25 μL PCR reaction contained 1.5 μl DNA, 1.5 units of Taq polymerase (Promega), 1x PCR buffer with 1.5 mM MgCl_2_, 1 mM each dNTP, and 0.8 μM of each primer. Amplification conditions were: 94°C for 1 min; 35 cycles of 94°C for 30s, 56°C for 30s, 72°C for 30s; 72°C for 2 min; 4°C hold. Amplifications were done in duplicate, and amplicons were visualized on 2% NuSieve gels (Cambrex, Rockland, ME).

**Table 2:** Primers used for characterization and real-time PCR

**Gene**	**Forward primer (5’-3’)**	**Reverse primer (5’-3’)**	**Reference**
16S	CGGGGAGGAAGGTGTTGTG	GAGCCCGGGGATTTCACATC	[38]
*hilA*	CGCTGGCAGAATGCTACCTC	AGCCCCAGTAATCCTAAAGCTTG	[39]
*prgH*	GCTCTTTCTTGCTCATCGT	ATCTCTATCTGGCTGGATACCT	This study
*invF*	ATGTGAAGGCGATGAGTAAC	GCTGCTGAATAGTGTAGAAGG	This study
*tetA*	GCTACATCCTGCTTGCCTTC	CATAGATCGCCGTGAAGAGG	[13]
*tetB*	GCTTTCAGGGATCACAGGAG	CCAAGACCCGCTAATGAAAA	[13]
*tetC*	GCATAAACCAGCCATTGAG	GGTAAACGCCATTGTCAG	This study
*tetD*	GATGTGGCGAATAAAGCG	CCAGTGTGACCCCTGTTAC	This study
*tetG*	CCTTGCAGGCAATGCTCTCAAACA	AGATTGGTGAGGCTCGTTAGCGTT	[40]
*thdF-S001*	GGGACGGATTTTCTCCAG	CGAGTTAGGGTTACGCTTG	This study
*S013*	CCACAATTTGGCTGTGATGGCTCA	TGGTCGTGTTATTAGCGGCCAGAT	[40]
*S044*	TGGACGCTCGAAGAGGTAGAG	AAGGATGGCCGTCGTCACT	[40]

### Culture conditions

For each experiment, *Salmonella* was plated on Lennox L (LB) agar plates (Invitrogen, Carlsbad, CA), and a single colony was selected and grown in LB broth with agitation for 6 hours at 37°C. A 1:1000 dilution of the 6 hour culture was made in LB broth and grown with agitation at 37°C overnight. A 1:200 dilution of the overnight culture was made in LB broth and divided into 16x100 glass tubes. Depending on the assay, the cultures were grown to either early-log (OD_600_ = 0.15) or late-log (OD_600_ = 0.6) with agitation before tetracycline addition.

### Growth curves

Growth curves for each isolate were determined by diluting overnight cultures 1:200, growing to early-log phase (OD_600_ = 0.15), and adding serial dilutions of tetracycline (0–256 μg/ml); this corresponds to the early-log growth phase to be tested, and was necessary to determine the effect of the antibiotic at this time point. Cultures were shaken continuously, and growth curve measurements (OD_600_) were taken every hour for 24 hours using a Bioscreen C instrument (Growth Curves LTD, Raisio, Finland). Differences between the no-antibiotic control and the other sample conditions during the logarithmic growth phase (0–9 hours) were determined by a one-way ANOVA with Dunnett’s post-test using GraphPad Prism 5 (GraphPad Software, San Diego, CA). *P* values less than 0.05 were considered significant.

### Experimental conditions

The effect of tetracycline during early-log growth phase was examined using overnight cultures that were diluted 1:200 in LB, subcultured into four tubes, and grown to OD_600_ = 0.15. An aliquot was taken for RNA analysis from each culture and placed in RNAProtect (QIAGEN, Germantown, MD). Tetracycline was then added to a final concentration of 0 (control), 1, 4, and 16 μg/ml to the four tubes for each isolate, and these were incubated with agitation at 37°C for 30 min (final OD_600_ = ~0.30). Aliquots for RNA analysis were taken from each bacterial culture and placed in RNAProtect. An additional aliquot was taken from each culture for a cell culture invasion assay. To test the effect of tetracycline during late-log growth phase, each overnight culture was diluted 1:200 in LB, split into four tubes, and grown to OD_600_ = 0.15. An aliquot was taken for RNA analysis from each culture and placed in RNAProtect. After these cultures grew to OD_600_ = 0.60, tetracycline was added to a final concentration of 0 (control), 1, 4, and 16 μg/ml to the four tubes for each isolate and incubated with agitation at 37°C for 30 min (final OD_600_ = ~0.70). Aliquots for RNA analysis were taken from each bacterial culture and placed in RNAProtect. An additional aliquot was taken from each culture for a cell culture invasion assay. All experiments were performed four separate times.

### *Salmonella* invasion assays

The aliquots taken following the 30 minute incubation with and without tetracycline were centrifuged at 16,000 x g for 2 minutes, and the pellets were re-suspended in fresh LB broth to remove the antibiotic. Invasion assays were performed with technical replicates for each biological replicate using a gentamicin protection assay in HEp-2 cells at a multiplicity of infection of ~40 as previously described [41]. Percent invasion was calculated by dividing CFU/ml recovered by CFU/ml added. The significance of the differences in invasion were determined by a one-way repeated measures ANOVA with Dunnett’s post-test to assess pair-wise differences between the no-antibiotic control and the other sample conditions using GraphPad Prism 5. *P* values less than 0.05 were considered significant. Each isolate had a different invasion rate without tetracycline, therefore invasion at 1, 4, and 16 μg/ml tetracycline was normalized to the control for each isolate at each growth phase for graphical representation of the fold change; the complete pre-normalized invasion data can be found in Additional file 1.

### Real-Time PCR assays

RNA was isolated using the RNeasy Mini Kit (QIAGEN, Germantown, MD), and genomic DNA was removed using the Turbo DNase DNA-free kit (Ambion, Austin, TX) according to the directions from the manufacturers. Total RNA was quantitated on a Nanodrop ND-1000 spectrophotometer (Thermo Scientific, Wilmington, DE). Reverse transcription was carried out using the Applied Biosystems High capacity cDNA reverse transcription kit on total RNA using random primers (Life Technologies, Grand Island, NY), and technical replicates were performed for each biological replicate. Real-Time PCR was performed in a Bio-Rad CFX96 Real-Time PCR Detection System (BioRad Laboratories, Hercules, CA) using the SYBR Green Master Mix (Applied Biosystems, Foster City, CA). Primer sets were used to evaluate the 16S *rRNA*, *hilA*, *prgH*, *invF*, *tetA*, *tetB*, *tetC*, *tetD*, and *tetG* transcripts (Table 2). For control assays, reverse transcriptase was not added to parallel mixtures for each sample. Amplification was performed using the following cycle conditions: 95°C for 10 min; 40 cycles of 95°C for 15 s, 55°C for 30 s, 72°C for 30 s; melting curve analysis from 65°C to 95°C. Raw data was analyzed using LinRegPCR software, and amplification efficiencies and cycle threhhold (C_T_) values were determined using a Window of Linearity for each primer set [42]. Expression differences were calculated by the Pfaffl method (Ratio = (E_target_)^ΔCTtarget(control-treated)^ / (E_reference_)^ΔCTreference (control-treated)^), where the 16S gene was the reference gene and the pre-antibiotic sample was the control condition for each isolate at each tetracycline concentration [43]. Values were log_2_ transformed, and GraphPad Prism 5 was used to perform a one-way repeated measures ANOVA with Dunnett’s post-test to assess pair-wise differences between the no-antibiotic control and the other sample conditions. *P* values less than 0.05 were considered significant. A heat map was constructed to display the differences in the real-time data relative to the control after tetracycline exposure; the numerical real-time data can be found in Additional file 1.

### Availability of supporting data

The data sets supporting the results of this article are included within the article and its additional file.

## Competing interests

The authors declare that they have no competing interests.

## Authors’ contributions

BWB conceived the study, and SMDB and BLB helped design it. BWB conducted the experiments. BWB, SMDB, and BLB analyzed and interpreted the data. BWB drafted the manuscript and SMDB and BLB helped revise it. All authors read and approved the final manuscript.

## Supplementary Material

Additional file 1: Table S1 Invasion and gene expression data. Four biological replicates were performed for each condition tested, and the table lists the average, standard error of the mean, and significance compared to the control. Each of the eight isolates (1434, 5317, 752, 1306, 4584, 290, 360, and 530) was tested at four different tetracycline concentrations (0, 1, 4, and 16 μg/ml) during two different growth phases (early- and late-log) for changes in invasion, as well as changes in gene expression at up to eight different loci (*hilA*, *prgH*, *invF*, *tetA*, *tetB*, *tetC*, *tetD*, *tetG*). Invasion data are listed as percentages, and the expression data are log_2_-fold changes. Significance is indicated for P < 0.05 (*), P < 0.01 (**), and P < 0.001 (***). Click here for file
